# Pathway from Exposure to an E-Cigarette Prevention Social Media Campaign to Increased Quitting Intentions: A Randomized Trial Among Young Adult E-Cigarette Users

**DOI:** 10.3390/ijerph22020307

**Published:** 2025-02-18

**Authors:** Alexander P. D’Esterre, Shreya Tulsiani, Elizabeth C. Hair, Madeleine Aseltine, Linda Q. Yu, Megumi Ichimiya, Jeffrey B. Bingenheimer, Jennifer Cantrell, W. Douglas Evans

**Affiliations:** 1 Schroeder Institute, Truth Initiative, Washington, DC 20001, USA; 2 Department of Health, Behavior, and Society, Johns Hopkins Bloomberg School of Public Health, Baltimore, MD 21205, USA; 3 Department of Prevention and Community Health, Milken Institute School of Public Health, The George Washington University, Washington, DC 20037, USA; 4 Global Institute of Public Health, New York University, New York City, NY 10003, USA

**Keywords:** e-cigarettes, tobacco, young adults, health campaign, campaign evaluation

## Abstract

In 2022, 26–31% of young adults reported using e-cigarettes in the previous 30 days. Research supports the effectiveness of mass media health campaigns in changing targeted attitudes and behaviors regarding nicotine use. However, the effect of social media campaigns and the pathway through which they change attitudes and behaviors require more research. This randomized controlled experiment examines the pathway through which exposure to an e-cigarette prevention social media campaign influences intentions to quit e-cigarettes among young adults who currently use e-cigarettes. Participants (*n* = 160) aged 18 to 24 years old were recruited through Virtual Lab in Facebook and Instagram. Structural equation modeling (SEM) was used to examine the pathway from campaign exposure, to changes in targeted attitudes, and finally to intentions to quit e-cigarettes in the next year. Ad exposure was significantly associated with stronger anti-industry attitudes, independence from e-cigarettes, and risk perceptions. These campaign-targeted attitudes were significantly associated with greater intentions to quit e-cigarettes (anti-industry attitudes (OR = 1.43), independence (OR = 1.50), and risk perception (OR = 1.71)). The findings suggest that exposure to an e-cigarette prevention social media campaign can affect targeted attitudes, which in turn improve intentions to quit. Future research should examine behavior changes and compare the effects between those currently using e-cigarettes and those not using them.

## 1. Introduction

E-cigarette use remains high among young adults, with 11% of young adults aged 18 to 24 years old reporting using e-cigarettes “every day” or “some days” in 2021 [1]. Other data reports have shown increases since then; in 2022, according to data from the Monitoring the Future survey, 26–31% of young adults reported utilizing e-cigarettes in the previous 30 days, which was nearly double the prevalence first reported in 2017 [2]. Because nicotine is a highly addictive substance and can harm the developing brain, the popularity of these products is concerning [3,4]. The use of e-cigarettes has also been linked to poor mental health outcomes, such as increased symptoms of anxiety and depression, sleep problems, and difficulty concentrating, remembering, or making decisions [5,6,7,8,9]. In addition to these impacts on mental health, e-cigarette use is also associated with physical health problems, including incident respiratory disease, COPD, and asthma [10,11].

Young adults’ exposure to tobacco-related environmental and media content is not only associated with lifetime, 30-day, and initiation of nicotine/tobacco use; this exposure also influences their perception of e-cigarettes and the use behaviors among peers [12,13]. Because of the evolving research around the dangers of youth e-cigarette use and the products’ continued popularity, it is critical to understand what influences youth e-cigarette use behavior. The theory of planned behavior (TPB) posits that attitudes, subjective norms, and perceived behavioral control affect behavioral intentions, which can then lead to behavior change [14]. The TPB framework has been instrumental in the development of impactful mass media health campaigns, including those aimed at influencing smoking prevention and cessation [15,16,17]. However, there have been fewer studies examining e-cigarette prevention media campaigns, and even fewer that focus on digital campaigns.

Recent research on e-cigarette prevention mass media campaigns has shown that they can impact e-cigarette knowledge, attitudes, and behaviors among youth and young adults. Hair et al. observed that those who reported high levels of awareness of the truth^®^ e-cigarette prevention campaign had stronger perceptions of product harm, the social unacceptability of e-cigarette use, and anti-industry sentiment than those with no awareness [18]. Other evaluations of nicotine prevention campaigns found that population-level awareness among youth and adults was associated with lower odds of intentions to use e-cigarettes and a dose-response decrease in the odds of using e-cigarettes [19,20]. Furthermore, previous research with current users of e-cigarettes has shown that reported awareness of an e-cigarette prevention campaign predicted changes in targeted e-cigarette attitudes, and those attitudes then predicted decreases in the frequency of e-cigarette usage [21]. These findings are further supported by a history of evaluation studies that show the effectiveness of health campaigns [22,23,24].

Hair and colleagues (2024) found, through structural equation modeling, that awareness of the truth^®^ e-cigarette prevention campaign follows a pathway in which it influences campaign-targeted attitudes, which in turn affect injunctive norms, ultimately reducing e-cigarette usage among young people [18]. Similarly, Kreslake et al. (2023) utilized structural equation modeling to show that current users of e-cigarettes who reported higher awareness of an e-cigarette prevention campaign displayed shifts in their attitudes toward e-cigarettes, which in turn predicted a subsequent decrease in e-cigarette utilization [21]. The current study builds on these previous observational studies by conducting a randomized controlled trial within users’ regular social media platforms. This study draws on experimental evidence gathered through social media-based data collection and is part of a larger series of randomized experiments reported elsewhere [25]. The purpose of this study is to examine the pathway through which intentions to quit e-cigarettes may be influenced by exposure to a relevant campaign among young adults. The campaign discussed in this study aimed to change the following attitudes: anti-industry attitudes, independence from e-cigarettes, and risk perceptions of e-cigarette use. We hypothesize that exposure to the ad campaigns will result in significant changes in the targeted attitudes, and that those attitudes will impact e-cigarette users’ intentions to quit e-cigarettes in the following year.

## 2. Materials and Methods

### 2.1. Study Design

The participants were recruited on Facebook and Instagram through the Virtual Lab platform and were provided with a survey through Facebook Messenger to measure baseline attitudes and demographics [26]. The Virtual Lab platform is an open-source tool that utilizes digital marketing, or “retargeting”, to recruit customized audiences of participants based on specific characteristics and online behavior. The recruited participants were eligible if they lived in the United States, were 18 to 24 years old, and had a Facebook or Instagram account. Virtual Lab ensures that all participant accounts are active and fit our recruitment criteria, and it provides testing to ensure that humans are filling out the survey at each wave. Informed consent was obtained from all of the individual participants included in the study. Only participants who reported the use of an e-cigarette at least once in the previous 30 days were included in the current study, as intentions to quit e-cigarette usage were not relevant for those who were not actively using e-cigarettes. This study, and the consent process, were reviewed and approved by the [REMOVED FOR REVIEW ANONYMITY] Institutional Review Board (IRB) on 5 August 2020 (IRB number NCR202837).

Upon completing the baseline assessment and providing informed consent, the eligible participants were then randomized to either the control or to one of the four treatment conditions. These conditions varied based on the maximum number of impressions presented to individuals and resulted in the following distribution: 0 (control), 4, 8, 16, or 32 impressions over a 60-day period. The participants who were randomized to the treatment conditions received the corresponding number of short videos directly into their Meta (i.e., Facebook and Instagram) social media feeds. These videos were pulled from a Truth Initiative marketing campaign called “Tested on Humans”, which conveyed the idea that e-cigarette companies are pushing products with unknown health risks and are, in effect, using their customers as test subjects. These videos were selected because they represented an anti-industry marketing strategy that has been shown to be effective in previous studies [25]; they targeted other validated attitudinal constructs (e.g., independence from e-cigarettes and risk perceptions) [27] and were not being aired during the time of this study. Previous research by Hair et al. (2023) has shown that this ad is effective at significantly influencing knowledge, beliefs, and attitudes regarding e-cigarette usage [28].

Following treatment randomization, two follow-up surveys were conducted to measure targeted attitudes and participant intentions to quit e-cigarettes following the completion of treatment randomization. The first survey was conducted 30 days after the participants were assigned to their respective conditions, and it asked the participants to report their attitudes regarding anti-industry beliefs, the social unacceptability of e-cigarette use, independence from e-cigarettes, and perceptions of the risk of harm of e-cigarette usage. A second survey was conducted 60 days after the initial assignment and was utilized to measure the intentions of the participants to quit using e-cigarettes within the next year. These surveys were delivered through Facebook Messenger by a pre-programmed chatbot.

While every effort was made to ensure that the participants in each study arm received the designated number of impressions, the number of actual impressions received differed slightly from the targeted values. The intended number of impressions per user in each arm was 0 (control), 4, 8, 16, and 32; however, the average number of impressions delivered per treatment arm was as follows: Arm 1 = 0, Arm 2 = 2.293, Arm 3 = 7.708, Arm 4 = 12.718, Arm 5 = 14.218. This discrepancy is likely due to the short gaps between waves when the participants were meant to receive these impressions—and because of this distribution, it was decided to combine the participants in Arm 4 and Arm 5 for our analyses. The treatment predictor variable was recoded to be ordinal, where Arm 1 was coded as “0”, Arm 2 as “1”, Arm 3 as “2”, and Arm 4 and 5 as “3”.

### 2.2. Measures

The primary outcome measure of interest was collected at the second follow-up and consisted of the participants’ reported intention to quit using e-cigarettes (“Are you seriously thinking about quitting e-cigarettes/vapes for good?”). The participants’ responses were treated as binary for the sake of these analyses, with the response “No, I am not thinking about quitting” coded as no intent, while the responses “Yes, but not within the year”, “Yes, within the year”, “Yes, within the next 6 months”, “Yes, within the next 30 days”, or “I’ve already quit” were coded as an intention to quit.

In addition to this measure of the intention to quit, we were also interested in the possibility that the effect of advertisement exposure on intentions to quit the use of e-cigarettes might be affected by attitudes related to e-cigarettes, such as anti-industry beliefs, social unacceptability of e-cigarette use, independence from e-cigarettes, and perceptions of the risk of harm of e-cigarette usage. The campaign also targeted these attitudinal constructs, except for social unacceptability. Therefore, we included several items for these attitudes.

The anti-industry beliefs, social unacceptability, and independence from e-cigarettes items were all measured on a six-point scale ranging from “Strongly Disagree” to “Strongly Agree”. The anti-industry attitudes measure was a composite made from the average of two items (“I am willing to stand up with others against vape companies” and “Vape companies make me angry”); the social unacceptability composite was an average of three items (“Vaping is okay to do socially with friends” (reverse coded), “Vaping is fine as long as you’re not addicted” (reverse coded), and “Vaping is not okay for people my age”); and the independence from e-cigarettes composite was an average of two items (“Not vaping is a way to show my independence” and “I am more in control of my life when I don’t vape”). The risk of harm composite was an average of three items rated on a 5-point scale ranging from “Very Unlikely” to “Very Likely” (“Feel bad physically because of vaping”, “Feel bad mentally or emotionally because of vaping”, and “Developing brain changes that affect the way you think because of vaping”). These scales were utilized due to their previous utilization in assessing the impact of e-cigarette prevention and cessation campaigns and to their validity and reliability [27].

### 2.3. Statistical Analysis

The relationship between the treatment, attitude, and intention questions were addressed using structural equation modeling (SEM) in Mplus 8.2. Utilizing SEM allowed us to accurately model the longitudinal nature of our data by tracing the impact of treatment assignment on participant attitudes 30 days after the onset of impressions, and the subsequent effect of those attitudes on the participants’ quitting intentions at 60 days. All covariates were measured at baseline and were included, as in the model for the first step, where treatment was the predictor of attitudes. The covariates included age category, gender, perceived financial status, and race and ethnicity.

## 3. Results

The characteristics for the young adult sample of current e-cigarette users (*n* = 160) are presented below in Table 1. The majority of the sample was female (73%). Nearly half identified as non-Hispanic White (48%), with the rest of the sample identifying as non-Hispanic Black (9%), Hispanic/Latino (21%), or another race/ethnicity (including multiracial) (23%). Most of the sample was aged 21–24 years old (63%). When asked about socioeconomic status, only a small percentage responded that they “don’t meet basic expenses” (7%), while the rest of the sample answered they “live comfortably” (33%), “meet needs with a little left” (34%), or “just meet basic expenses” (27%). All of these demographic variables were controlled for by entering them as the first step in the model as predictors of treatment condition; however, education was excluded as a predictor as it was found to be highly collinear with age for this sample. For all four models, none of the covariates was significant, indicating that randomization was successful and that all demographic groups were represented evenly across all levels of treatment. The control and each of the four treatment arms were supposed to consist of 20% of the sample, but attrition and the combining of Arms 4 and 5 led to a slight deviation from this design. Ultimately, 21.88% of the sample were in the Arm 1 (control) condition, 20.62% in Arm 2, 13.75% in Arm 3, and 43.75% in Arm 4/5.

Overall, the results of SEM showed that campaign exposure had a significant impact on the campaign-targeted anti-industry attitudes, risk perceptions, and independence. The mean values of all four attitudes at the baseline assessment were slightly above the midpoint for the respective measures, indicating mild agreement with the anti-industry attitudes, independence from e-cigarettes, and social unacceptability belief measures, as well as with the belief that e-cigarettes were slightly more likely than not to cause a risk of harm—even before being exposed to the intervention. However, the results of the first step of the model illustrate how agreement with these campaign-targeted attitudes increased further with additional exposure to the campaign. All attitudinal measures were assessed for nonnormality utilizing the Shapiro–Wilk test of normality prior to inclusion in our models, and no evidence of significant deviation from normality was found (anti-industry: *p* = 0.832; risk perception: *p* = 0.620; independence: *p* = 0.997; social unacceptability: *p* = 0.199).

**Figure 1:**
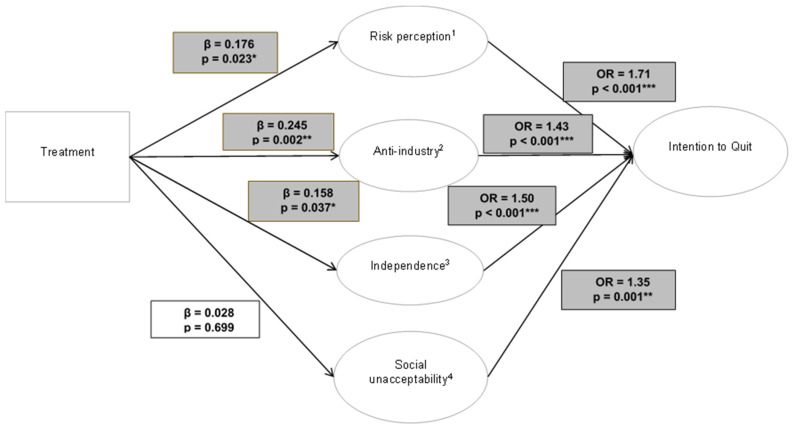
Pathways from treatment of campaign exposure to intentions to quit e-cigarettes among young adults who currently use e-cigarettes, *n* = 160. Notes: 1 = perceived risk of e-cigarette harm; 2 = anti-industry sentiments; 3 = desire for independence from e-cigarettes; 4 = affinity for groups that reject e-cigarette use. * *p* < 0.05. ** *p* < 0.01. *** *p* < 0.001.

Figure 1 illustrates the pathway from the treatment of campaign exposure to the intentions to quit e-cigarettes and the influence of the campaign-targeted attitudes. The results of SEM show that ad exposure had a positive effect on the sample’s alignment with the campaign-targeted risk perception (β = 0.176, *p* = 0.023), anti-industry (β = 0.245, *p* = 0.002), and independence (β = 0.158, *p* = 0.037) attitudes. However, there was not a significant association between treatment and social unacceptability (β = 0.028, *p* = 0.699), which was the only attitude measured but which had not been targeted by the campaign. As seen in the next step in Figure 1, greater alignment of every attitudinal construct was associated with an increased likelihood of intending to quit e-cigarettes, as indicated by the odds ratios (OR) greater than 1 (risk perception (OR = 1.71, *p* < 0.001), anti-industry (OR = 1.43, *p* < 0.001), independence (OR = 1.50, *p* < 0.001), and social unacceptability (OR = 1.35, *p* = 0.001)). More information regarding each model can be found in Supplementary Materials.

## 4. Discussion

The findings from this study support recent research showing that exposure to an e-cigarette prevention social media campaign can improve targeted attitudes and intentions to quit e-cigarettes among young people who currently use e-cigarettes [19]. This study stands out for its high external validity due to its experimental design of a campaign specifically implemented on social media, which is an influential medium for reaching young populations [29]. Our findings support those of a recent similar longitudinal study that observed behavior changes from an e-cigarette prevention digital media campaign in its 3-year study period [18].

The resulting pathway from this two-month study is consistent with the theory of planned behavior (TPB), demonstrating a stepwise approach for e-cigarette prevention campaigns. TPB states that changes in attitudes, social norms, and perceived control over the behavior (e.g., independence from e-cigarettes) influence intentions to change the behavior, which eventually result in actionable behavior change [14]. The significant changes in intentions observed after a short exposure and the subsequent change in stated intentions provide reasons to suggest that even a short campaign may result in changes in behavior. However, future research directly measuring e-cigarette usage behavior and quitting attempts following this exposure would be better able to speak to the long-term impact of this type of intervention, and subsequent studies would benefit from an additional follow-up 6 months or 1 year after campaign exposure.

These results show that ad exposure had the greatest effects on risk perception, independence from e-cigarettes, and anti-industry attitudes. As expected, there was no significant association between the treatment and the social unacceptability of e-cigarette usage, as it was not an attitude targeted by the campaign. These increases in attitudinal endorsement were significantly associated with increased intentions to quit e-cigarettes. The findings of this study further support the importance of e-cigarette prevention social media campaigns that target attitudes that have been validated in terms of their influence on intentions and behavior [27] and suggest that social media prevention campaigns may provide an avenue to create significant real-world changes in cessation intentions.

Exposure to nicotine and tobacco content on social media is a risk factor for use, especially among young adults [30]. This study has implications for future interventions and research to counteract these influences using social media. Our findings add to the emerging evidence that social media interventions are effective in changing nicotine and tobacco use behaviors [31]. Future research should explore changes in the observed mediators from this study over time and examine dose-response effects. Future interventions should be designed to influence the mediators observed in this research and their effects on e-cigarette usage and/or quitting attempts among young adults. Studies should also examine which message strategies, such as highlighting the mental health effects of e-cigarettes, are the most effective in changing the observed mediators of e-cigarette use.

### Limitations

While the study described within this manuscript has significant real-world implications, there are several areas in which future studies could expand upon these findings. First, the study period described was 60 days, but previous research has shown that behavior change can take 18 months after exposure to a media campaign [32]. The current study has shown significant changes in intentions following a short exposure, but it is less clear how well those changes in intentions will result in changes in behavior. While previous studies suggest that intentions to quit nicotine use do significantly predict future quitting attempts [33,34], the field would benefit from additional research on this topic. To accomplish this task, future studies would benefit from a longer-term longitudinal design, to allow time for changes in behavior to occur. Second, the current focus of this paper is on cessation for current users of e-cigarettes; thus, these results cannot speak directly to the effectiveness of such an approach when considering e-cigarette prevention. Further research is necessary to assess the effectiveness of such an approach with a nicotine-naïve sample. Additionally, the impression data provided by Meta are aggregated, meaning that an individual’s survey responses cannot be tied back to the actual number of impressions to which they were exposed. Thus, we cannot examine the effect of the number or frequency of impressions more thoroughly, nor what the impact is based on the platform of exposure (e.g., Facebook or Instagram). This data aggregation, however, reflects the real-life limitations of how Meta provides data to advertisers. Finally, the participants were recruited with consideration given to providing various demographic groups with representation, but a complex survey methodology was not employed; thus, the generalizability of these findings is somewhat restricted.

## 5. Conclusions

This study provides evidence for a pathway by which an e-cigarette prevention campaign on social media may influence intentions related to e-cigarette cessation. Exposure to the campaign significantly improved campaign-targeted attitudes, which then increased intentions to quit e-cigarettes among current young adult users of e-cigarettes. This study also highlights gaps in the literature: future research should assess the effects of a social media campaign on behavior through a significantly longer follow-up procedure and should compare the effects of these ads on current and non-current users of e-cigarettes.

The results of this study showcase the potential effectiveness of e-cigarette prevention ads when inserted directly into the social media feeds of young adults. Policymakers and public health groups with an interest in promoting e-cigarette cessation may be able to utilize a similar approach to reach their target population and generate significant shifts in attitudes and intentions.

## Figures and Tables

**Table 1:** Sample characteristics for participants who currently use e-cigarettes (*n* = 160).

Demographic Variable	*n* (%)
**Treatment Groups**	
0	35 (21.88%)
1	33 (20.62%)
2	22 (13.75%)
3	70 (43.75%)
**Age**	
18–20	60 (37.50%)
21–24	100 (62.50%)
**Education**	
Less than High School	31 (19.38%)
High School	82 (51.25%)
Bachelor’s or Greater	43 (25.88%)
Missing/Refused	4 (2.50%)
**Gender**	
Female	116 (72.50%)
Male	41 (25.62%)
Non-binary/different identity	3 (1.88%)
**Perceived Financial Status**	
Do not meet basic expenses	11 (6.88%)
Just meet basic expenses	43 (26.88%)
Meet needs with a little left	54 (33.75%)
Live comfortably	52 (32.50%)
**Race/Ethnicity**	
Non-Hispanic White	77 (48.12%)
Non-Hispanic Black	14 (8.75%)
Hispanic/Latino	33 (20.62%)
Another Race/Ethnicity, including Multiracial	36 (22.50%)
**Means for Attitudes (Baseline)—mean (SD)**	
Anti-Industry	3.36 (1.01)
Risk Perception	3.11 (1.16)
Independence from E-Cigarettes	3.39 (0.97)
Social Unacceptability	3.62 (0.96)

The bolded text are subheadings explaining the non-bolded text in the next set of rows. (e.g., 0, 1, 2, and 3 are all “Treatment Groups”).

## Data Availability

The study was registered as a clinical trial at clinicaltrials.gov under identifier NCT04867668. A data-sharing agreement is required for use of all data, analytic code, and other materials used in this study. Truth Initiative does not share any of these three with tobacco industry representatives or affiliated researchers. Investigators seeking access to data, code, and materials used in the study should make a written request to the lead author and submit a detailed research plan including the purpose of the proposed research, required variables, duration of the analysis phase, and IRB approval with FWA information and documentation of investigator training in human subjects. Approved investigators may access datasets, analytic code, and other materials via an analytic portal owned and administered by Truth Initiative.

## Supplementary Materials

The following supporting information can be downloaded at: https://www.mdpi.com/article/10.3390/ijerph22020307/s1, Table S1: Model Fit Information and Regression Coefficients.
