# Supplementary material for: Pathway from Exposure to an E-Cigarette Prevention Social Media Campaign to Increased Quitting Intentions: A Randomized Trial Among Young Adult E-Cigarette Users
Source: Int J Environ Res Public Health. 2025 Feb 18;22(2):307. doi: 10.3390/ijerph22020307 (PMC11855058; doi:10.3390/ijerph22020307)
Supplement: Supplementary file 1 [file ijerph-22-00307-s001.zip › ijerph-3410718-supplementary.pdf]

Supplementary Table 1. Model Fit Information and Regression Coefficients

	Risk Perception	Anti-Industry	Independence	Social Unacceptability
Model Fit Information:				
RMSEA	0.015	0.000	0.006	0.026
CFI	0.968	1.000	0.993	0.674
Chi-Squared Model Fit (p-value)	19.648 (0.416)	16.805 (0.603)	19.092 (0.451)	20.979 (0.338)
Effect of Demographics on Treatment Group:				
Age				
18-20	REF	REF	REF	REF
21-24	-0.022 (0.081)	-0.008 (0.082)	-0.010 (0.080)	-0.025 (0.080)
Gender				
Female	REF	REF	REF	REF
Male	-0.101 (0.086)	-0.126 (0.088)	-0.102 (0.086)	-0.123 (0.084)
Non-binary / Different Identity	-0.027 (0.080)	-0.043 (0.077)	-0.017 (0.082)	-0.018 (0.098)
Perceived Financial Situation				
Don't meet basic expenses	REF	REF	REF	REF
Just meet basic expenses	0.146 (0.171)	0.149 (0.170)	0.091 (0.166)	0.123 (0.167)
Meet needs with a little left	0.172 (0.181)	0.197 (0.181)	0.137 (0.175)	0.159 (0.177
Live comfortably	0.172 (0.180)	0.190 (0.180)	0.127 (0.171)	0.144 (0.175)
Race / Ethnicity				
Non-Hispanic White	REF	REF	REF	REF
Non-Hispanic Black	0.010 (0.082)	0.009 (.083)	0.029 (0.081)	0.030 (0.082)
Hispanic / Latino	-0.047 (0.085)	-0.029 (0.088)	-0.028 (0.084)	-0.024 (0.084)
Another Race / Ethnicity	0.117 (0.099)	0.117 (0.100)	0.145 (0.096)	0.152 (0.094)
Effect of Treatment Group on Measured Attitude:	0.176 (0.078) *	0.245 (0.079) **	0.158 (0.076) *	0.028 (0.071)
Effect of Measured Attitude on Quit Intentions:	0.536 (0.077) ***	0.359 (0.100) ***	0.403 (0.079) ***	0.303 (0.091) **
